# A Facile and Flexible Approach for Large-Scale Fabrication of ZnO Nanowire Film and Its Photocatalytic Applications

**DOI:** 10.3390/nano9060846

**Published:** 2019-06-02

**Authors:** Qingyang Li, Qiwei Wang, Zaijun Chen, Quanxin Ma, Maozhong An

**Affiliations:** 1 Institute of Advanced Wear & Corrosion Resistant and Functional Materials, Jinan University, Guangzhou 510632, China; qingyang@jnu.edu.cn; 2 Key Laboratory of Power Battery and Materials, School of Materials Science and Engineering, Jiangxi University of Science and Technology, Ganzhou 341000, China; 13097332709@163.com; 3 State Key Laboratory of Urban Water Resource and Environment, School of Chemistry and Chemical Engineering, Harbin Institute of Technology, Harbin 150001, China

**Keywords:** ZnO nanowires, nanocrystalline zinc, electrodeposition, hydrothermal treatment, photocatalytic activity

## Abstract

A novel strategy for large-scale synthesis of ZnO nanowire film is reported, which inherits the advantages of the solution-phase method and seeded growth process, such as low-temperature, efficient, economical, facile and flexible. It is easy to implement on various metals through room-temperature electrodeposition, followed by hydrothermal treatment at 90 °C, and suitable for industrialized production. The ZnO nanowires with an average wire diameter about 40 nm are in situ grown from and on nanocrystalline zinc coating, which forms a strong metallurgical bonding with the substrates. The p-type ZnO nanowire film has a well-preferred orientation along the (100) direction and a wurtzite structure, thereby displaying an effective photocatalytic capability for carcinogenic Cr^6+^ ions and CO_2_ greenhouse gas reduction under visible light irradiation. In addition to these features, the ZnO nanowire film is easy to recycle and, therefore, it has broad application prospects in contaminant degradation and renewable energy.

## 1. Introduction

ZnO nanowire film has recently attracted considerable attention in the fields of nanoscale electronics [1], photonics [2], gas sensors [3], solar cells [4], and photocatalysts [5], due to its unusual physicochemical properties, such as a wide bandgap, strong exciton binding energy and excellent thermal stability [6]. So far, many methods have been developed to synthesize ZnO nanowire film, typically described as chemical vapor deposition [7], vapor phase transport [8], physical vapor deposition [9], pulsed laser deposition [10], magnetron sputtering [11], thermal evaporation [12], thermal oxidation [13], electrodeposition (template-assisted or oxygen gas-assisted) [14,15], and hydrothermal reaction (or named solution-phase reaction) [16,17]. Among the above-mentioned approaches, the hydrothermal reaction is considered as one of the most promising processes for the economical synthesis of ZnO nanowire film, because it does not require a vacuum, high growth temperature, sophisticated equipment, precious metal catalysts, nano-template, and continuous gas injection [18]. In this regard, Vayssieres [19] produced the film by the natural adsorption of ZnO nanowire powder on the substrate from a solution containing Zn^2+^ ions at 95 °C. Yang et al. [20] directly synthesized ZnO nanowire film on various substrates from the same solution at 90 °C, with the help of a pre-spin-coated ZnO nanocrystal seed layer, and the method was expected to improve the binding force between the film and the substrate due to the use of a binder. In our earlier work, we demonstrated that the ZnO nanowires could also grow from nanocrystalline zinc [21]. Here we further expand this method to prepare ZnO nanowire film in situ on various metals through electrodeposition of the nanocrystalline zinc coating, followed by a mild hydrothermal treatment. The cost-effective and easy operated pre-deposition step cannot weaken the competitive advantage of flexible production of the seeded growth process, instead of further improving the film’s binding force, because the coating presents strong metallurgical bonding with the matrix metals. Meanwhile, the structure, composition, growth mechanism and performances including the lattice vibrational feature, semiconductor type and light absorption of ZnO nanowire film are discussed in detail. The present study especially focuses on its photocatalytic activity and the underlying mechanism.

## 2. Materials and Methods

**Figure 1:**
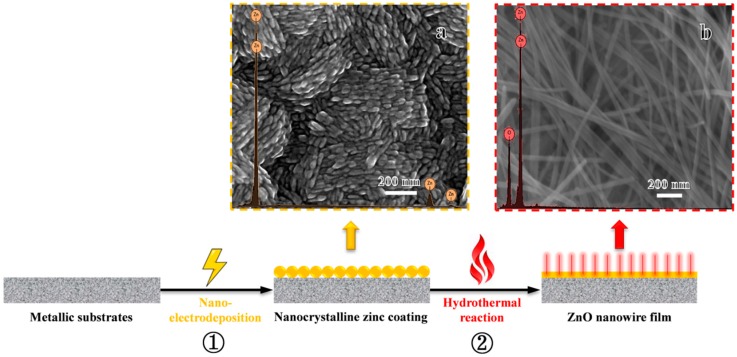
Schematic illustration of the synthetic procedure with scanning electron microscope (SEM) images and energy dispersive X-ray spectroscopy (EDXS) spectra of nanocrystalline zinc coating (**a**) as well as ZnO nanowire film (**b**).

The synthetic route of ZnO nanowire film is illustrated in Figure 1. Firstly, a nanocrystalline zinc coating was electrodeposited on the metallic substrates, i.e., a steel sheet (2.5 cm × 2 cm), from a sulfate bath containing 100 g L^−1^ zinc sulfate, 20 g L^−1^ boric acid and 1 g L^−1^ polyacrylamide with a pH of 1–2 (Figure 1, Step ①). The current density, deposition time and temperature were 3 A dm^−2^, 30 min and 25 °C, respectively. Secondly, the coating was rinsed with deionized water, followed by a hydrothermal process in 10 g L^−1^ sodium hydroxide and 20 g L^−1^ trisodium citrate dihydrate solution for 10 h at 90 °C (Figure 1, Step ②). After the above procedures, the ZnO nanowire film was immediately rinsed and dried, then subjected directly to structure and performance characterizations. The detailed test methods can be found in the Supporting Information with respect to the measurement methods.

## 3. Results and Discussions

**Figure 2:**
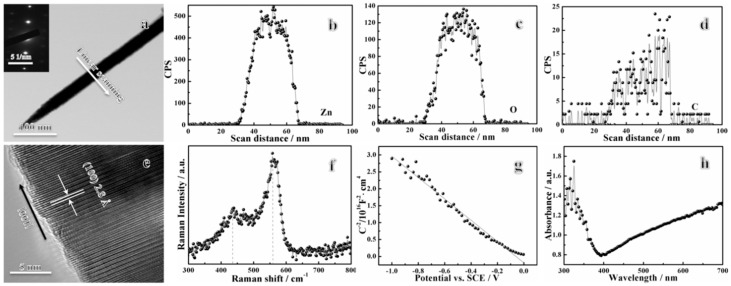
Transmission electron microscopy (TEM) image (**a**), selected-area electron diffraction (SAED) pattern (inset image of figure **a**), EDXS line-scan profiles (**b**) Zn, (**c**) O and (**d**) C, and high-resolution transmission electron microscopy (HRTEM) image (**e**) of single ZnO nanowire, Raman spectrum (**f**), Mott–Schottky plot (**g**) as well as UV-visible absorption spectrum (**h**) of ZnO nanowire film. The arrows in Figure 2a,e indicate the line-scan direction of EDXS and the preferred growth orientation of single ZnO nanowire, respectively.

The scanning electron microscope (SEM) images and energy dispersive X-ray spectroscopy (EDXS) spectra of nanocrystalline zinc coating and ZnO nanowire film are shown in Figure 1. A summary of the element compositions for both samples is listed in Table S1. It can be seen that the nano-grains (<50 nm) composed of pure zinc were approximately rice-shaped (Figure 1a). The nanocrystalline coating was adherent to the substrate and appeared to be defect-free, which a thickness of about 19.6 μm, according to the cross-sectional morphology (Figure S1). Furthermore, the X-ray diffraction (XRD) spectrum illustrated that the nanocrystalline zinc had a (110) preferred orientation (Figure S2), and its average grain size was about 33.7 nm (Table S2) calculated using Scherrer’s formula. After hydrothermal aging, a layer of nanowires with very homogeneous wire diameter distributions grew in situ on the nanocrystalline zinc coating (Figure 1b). The Zn:O atomic ratio in the nanowires was approximately a perfect 1:1 (Table S1), indicating that the nanowire film is ZnO. The transmission electron microscopy (TEM) image (Figure 2a), EDXS line-scan profiles (Figure 2b–d) and corresponding mappings (Figure S3) of a single nanowire also verified that the nanowires were ZnO, with a small quantity of carbon-doping, and the wire diameter was around 40 nm. Moreover, the selected-area electron diffraction (SAED) pattern in the inset of Figure 2a indicated that the ZnO nanowires were single-crystalline. The accompanying high-resolution transmission electron microscopy (HRTEM) image provided further structural details of the ZnO nanowires, in which the lattice spacing of ~0.280 nm between adjacent lattice planes corresponded to the distance between two (100) crystal planes, demonstrating that the ZnO nanowires exhibited a preferred growth orientation along the (100) plane (Figure 2e). It should be noted that the method is not limited to steel. The ZnO nanowire film can also be grown on various metals, such as copper, brass, zinc, and nickel, etc., through pre-electrodeposition of nanocrystalline zinc followed by a mild hydrothermal reaction (Figure S4), therefore, it is a flexible preparation method.

The ZnO nanowires grew directly from the nanocrystalline zinc, and what could be inferred from the phenomenon was that the wire diameter of ZnO was approximately equal to the grain size of zinc. The in situ growth process possibly involves the following reactions [22,23,24,25]:

(1) $$Zn+H_{2}O\to ZnO+H_{2}$$

(2) $$Zn+1/2O_{2}\to ZnO$$

(3) $$Zn+2OH^{-}\to ZnO+H_{2}O+2e^{-}$$

For instance, the H_2_O, O_2_ or HO^−^ may oxidize metallic Zn to ZnO. In order to decipher the growth mechanism exactly, the hydrothermal reaction of the nanocrystalline zinc coating in pure water was also investigated. The aerated aqueous solution contains H_2_O and O_2_, which provide the main reaction conditions of Equation (1) and Equation (2), respectively. There were not ZnO nanowires growing on the coating under the same temperature, suggesting that the actual reaction is Equation (3). Obviously, this growth mechanism is different from that of solution-phase reaction (Equation (4)) and seeded growth process (Equation (5)) of ZnO nanowire film produced from the following reactions [19,20]:

(4) $$Zn^{2+}+2OH^{-}\to Zn{\left(OH\right)}_{2}\to ZnO+H_{2}O$$

(5) $$ZnO_{\left(Nanoparticles\right)}\overset{Hydrothermal\;reaction}{\to }ZnO_{\left(Nanowires\right)}$$

In the abovementioned processes, the ZnO nanowire films grow from Zn^2+^ ions and ZnO nanoparticles, respectively.

Figure 2f depicts that the Raman spectrum of the ZnO nanowire film exhibited only E_2_ and abnormal volume phonon vibration modes at about 440 and 555 cm^−1^, respectively, revealing that the film had a wurtzite structure [26,27]. Furthermore, the Mott–Schottky result confirmed that the ZnO nanowire film is p-type semiconductor. According to the Mott–Schottky theory, the n-type and p-type semiconductors have a positive and negative slope of the linear $C_{SC}^{-2}\sim E$, respectively, as illustrated in the following equations [28].

The n-type semiconductor:

(6) $$\frac{1}{C_{SC}^{2}}=\frac{2}{\varepsilon \varepsilon_{0}N_{d}}(E-E_{fb}-\frac{kT}{e})$$

The p-type semiconductor:

(7) $$\frac{1}{C_{SC}^{2}}=-\frac{2}{\varepsilon \varepsilon_{0}N_{a}}(E-E_{fb}-\frac{kT}{e})$$

where *C*_SC_ is the capacitance value, *ε* is the dielectric constant of ZnO (8.5), *ε*_0_ is the vacuum permittivity (8.85 × 10^−14^ F cm^−1^), *N*_d_ and *N*_a_ are the carrier concentrations, *E* is the applied potential, *E*_fb_ is the flat band potential, *k* is the Boltzmann constant (1.38 × 10^−23^ J K^−1^), *T* is the absolute temperature, and *e* is the elementary charge (1.602 × 10^−19^ C). In our case, it is obvious that a negative linear relationship of $C_{SC}^{-2}\sim E$ plot is shown in Figure 2g.

The UV-visible (UV-vis) absorption spectrum also indicated that the ZnO nanowire film showed strong light absorption in both the ultraviolet region and visible region (Figure 2h). It is well-known that the bandgap of ZnO is approximately 3.4 eV. This means that ZnO exhibits absorption of photons only in the ultraviolet region. Therefore, the light absorption of the ZnO nanowire film in the visible region may come from the carbon doping, as shown in Figure 2d and Figure S3. The variation of (*αhv*)^2^ versus *hv* of the ZnO nanowire film is further plotted in Figure S5, from which the band gap of the sample could be estimated to be about 3.25 eV, based on Butler’s formula (Equation (8)) [29]:

(8) $${\left(\alpha hv\right)}^{2}=D(hv-E_{g})$$

where *α* is the optical absorption coefficient, *hv* is the photon energy (eV), D is a constant, and *E*_g_ is the band gap.

**Figure 3:**
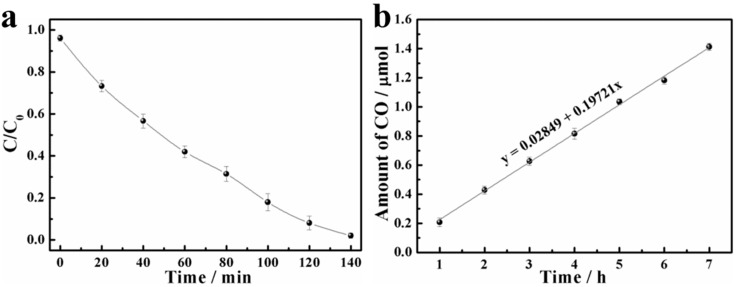
Photocatalytic Cr^6+^ ions (**a**) and CO_2_ (**b**) reduction of ZnO nanowire film under visible light irradiation.

As one of the most promising photocatalysts, the catalytic activity of the ZnO nanowire film in the photoreduction of Cr^6+^ ions under visible light irradiation was examined. Figure 3a illustrates the kinetic curve of Cr^6+^ photoreduction. It was clearly observed that the highly carcinogenic Cr^6+^ was almost completely reduced to low-toxic Cr^3+^ within 140 min. Considering that the solution volume decreased during continuous sampling, while the ZnO nanowire film was unconsumed, the photocatalytic degradation rate of Cr^6+^ in a 100 mL solution exposed to visible light for 140 min was also evaluated (Figure S6). The actual photoreduction rate of Cr^6+^ was about 97%, indicating that the effect of the sampling methods on experimental results was negligible. The stability of the ZnO nanowire film was investigated using a recycling experiment of Cr^6+^ reduction (Figure S7). After five cycles, the photoreduction rate did not show a distinct loss. The reaction mechanism can be expressed by the following equations:

(9) $$ZnO+hv\to ZnO(h^{+}+e^{-})$$

(10) $$h^{+}+C_{6}H_{8}O_{7}\to CO_{2}+H_{2}O$$

(11) $$h^{+}+H_{2}O\to •OH+H^{+}$$

(12) $$C_{6}H_{8}O_{7}+•OH\to CO_{2}+H_{2}O$$

(13) $$Cr_{2}O_{7}^{2}{}^{-}+14H^{+}+6e^{-}\to 2Cr^{3+}+7H_{2}O$$

(14) $$CrO_{4}^{2}{}^{-}+8H^{+}+3e^{-}\to Cr^{3+}+4H_{2}O$$

Briefly, the hole-electron (*h*^+^-*e*^−^) pairs were generated in the valence band and the conduction band of the ZnO nanowires upon irradiation with visible light (Equation (9)), respectively. The *h*^+^ could be scavenged by the organic acids (citric acid, C_6_H_8_O_7_) directly (Equation (10)) or indirectly (Equations (11) and (12)), thereby forming H_2_O and CO_2_, while the *e*^−^ could reduce Cr^6+^ to Cr^3+^ (Equations (13) and (14) depend on the adopted Cr^6+^ source) [30,31,32,33,34]. The photocatalytic activity of the ZnO nanowire film towards reduction of Cr^6+^ under visible light irradiation was better than that of the nanorods film counterpart [35], the nanoparticles powder counterpart [36] and other existent types of photocatalysts [37,38], such as TiO_2_ (nanoparticles powder), CuO (microflower film), SnO_2_ (nanoparticle powder), SnS_2_ (nanoflake powder) as well as g-C_3_N_4_ (microparticle film), as shown in Table S3 of the Supporting Information.

In addition to the photocatalyzed reduction of Cr^6+^, the photocatalytic capability of the ZnO nanowire film to convert CO_2_ selectively to CO was evaluated. As shown in Figure 3b, the conversion amount of CO was about 0.2 μmol h^−1^ (6.6 μmol m^−2^ min^−1^) via a reverse water-gas shift reaction, as represented by the following equation [39]:

(15) $$CO_{2}+H_{2}↔CO+H_{2}O$$

The 35 h consecutive test demonstrated that the ZnO nanowire film also exhibited optimum stability for CO_2_ photoreduction (Figure S8). To our knowledge, there has been no previous work published in the open literature on the photocatalytic conversion of CO_2_ to CO using pure ZnO under visible light irradiation. However, the photocatalytic activity of the ZnO nanowire film produced in this work was clearly better than that of CeO_2_ (nanorod, nanoparticle and nanocube powder correspond to 0.8, 1.1 and 2.0 μmol m^−2^ min^−1^ of CO production rate, respectively) reported in literature [40], demonstrating that the synthesized ZnO nanowire film displayed good and effective photoreduction activity to CO_2_. More importantly, the advantages of the ZnO nanowire film also include its easy recycling, in comparison to other powder-like counterparts. As shown in Figure S9, the ZnO nanowire film could be easily recycled and had no obvious shedding after the photocatalytic tests.

## 4. Conclusions

In summary, we developed a simple, flexible and cost-effective strategy, i.e., electrodeposition of nanocrystalline zinc coating followed by a mild hydrothermal treatment, for large-scale growth of ZnO nanowire film in situ on various metals. The ZnO nanowires directly grew from nano-Zn, which is different from the growth mechanisms of the solution-phase reaction and seeded growth process, which grow from Zn^2+^ ions and ZnO nanoparticles, respectively. The as-synthesized ZnO nanowire film was a p-type semiconductor with a well-preferred orientation along the (100) direction, with a homogeneous wire diameter distribution of around 40 nm and a wurtzite structure, which made it possible to exhibit an effective photocatalytic activity towards both Cr^6+^ ions and CO_2_ reduction under visible light irradiation. The ZnO nanowire film also had an easy to recycle feature, which is especially suitable for industrial applications.

## Supplementary Materials

The following are available online at https://www.mdpi.com/2079-4991/9/6/846/s1, Measurement methods, Figure S1: Cross-sectional morphology of nanocrystalline zinc coating, Figure S2: XRD spectrum of nanocrystalline zinc coating, Figure S3: TEM image (a) and corresponding EDXS mappings (b–d) of single ZnO nanowire, Figure S4: Digital photographs of steel, copper, brass, zinc as well as nickel substrates before (a–e) and after (a′–e′) coating of ZnO nanowire film, Figure S5: Variation of (*αhv*)^2^ versus the photon energy (*hv*) of ZnO nanowire film, Figure S6: Comparison of the photocatalytic degradation rate between different Cr^6+^ solutions exposed to visible light for 140 min in the presence of ZnO nanowire film: (a) a 100 mL solution after 7 continuous samplings (the sample interval is 5 mL every 20 min), and (b) a freshly prepared 100 mL solution, Figure S7: Photocatalytic activity of ZnO nanowire film for Cr^6+^ ions reduction with five separate cycles, Figure S8: Cycling curves of photocatalytic CO_2_ reduction for ZnO nanowire film, Figure S9: Digital photograph of ZnO nanowire film after the test of CO_2_ photoreduction, Table S1: A summary for the elemental content of different samples, Table S2: The grain size and texture coefficient calculated from XRD spectrum of nanocrystalline zinc coating, Table S3: Comparison of the Cr^6+^ ions reduction between ZnO nanowire film and other photocatalysts under visible light irradiation in literature.
