# Supplementary material for: A Facile and Flexible Approach for Large-Scale Fabrication of ZnO Nanowire Film and Its Photocatalytic Applications
Source: Nanomaterials (Basel). 2019 Jun 2;9(6):846. doi: 10.3390/nano9060846 (PMC6630657; doi:10.3390/nano9060846)
Supplement: Supplementary file 1 [file nanomaterials-09-00846-s001.pdf]

Supporting Information

A Facile and Flexible Approach for Large-Scale Fabrication of ZnO Nanowire Film and Its Photocatalytic Applications

Qingyang Li [1], Qiwei Wang [1,*], Zaijun Chen [2], Quanxin Ma [2,*] and Maozhong An [3,*]

1 Institute of Advanced Wear & Corrosion Resistant and Functional Materials, Jinan University, Guangzhou 510632, China; qingyang@jnu.edu.cn
2 Key Laboratory of Power Battery and Materials, School of Materials Science and Engineering, Jiangxi University of Science and Technology, Ganzhou 341000, China; 13097332709@163.com
3 State Key Laboratory of Urban Water Resource and Environment, School of Chemistry and Chemical Engineering, Harbin Institute of Technology, Harbin 150001, China
* Correspondence: wangqiwei@jnu.edu.cn (Q.W.); 9120170087@jxust.edu.cn (Q.M.); mzan@hit.edu.cn (M.A.)

Measurement methods

The surface and cross-sectional morphologies, element composition, as well as crystal texture of nanocrystalline zinc coating and ZnO nanowire film, were characterized by scanning electron microscope (SEM, Helios Nanolab 600i) with energy dispersive X-ray spectroscopy (EDXS), transmission electron microscopy (TEM, JEOL JEM-2010) and X-ray diffraction (XRD, Rigaku Corporation Dmax-3B). Raman scattering spectrum (Renishaw inVia) was conducted to investigate the lattice vibrational property of ZnO nanowire film using a 458 nm excitation source from an argon ion laser. The Mott–Schottky test was carried out using CHI 760E electrochemical workstation with a standard three-electrode system. The ZnO nanowire film, saturated calomel electrode (SCE), platinum plate and 0.5 M Na_2SO_4 solution were used as the working, reference, auxiliary electrode and electrolyte, respectively. The plot was obtained by using a 10 mV sine wave modulated signal with a constant frequency of 1 kHz and step rate of 5 mV. A UV-visible spectrophotometer (Hitachi, U-3010) was used to determine the absorbance performance of ZnO nanowire film.

The photocatalytic reduction of the Cr^{6+} ions was performed in a quartz reactor containing the ZnO nanowire film and 100 ml Cr^{6+} solution (10 mg L^{-1}) under visible light irradiation. A 300 W xenon arc lamp (Perfect Light PLS-SXE300) with a filter (λ > 400 nm) was served as the light source. In a typical process, the ZnO nanowire film was immersed in the solution for 30 min in dark to establish an adsorption-desorption equilibrium. Then, 0.05 g citric acid was added to the above system, and 5 ml solution was collected every 20 min for ultraviolet-visible spectrophotometer (HITACHI UH-5300) test. The concentration of Cr^{6+} was determined by monitoring the absorbance variation at 350 nm, thereby calculating the reduction rate. The Cr^{6+} photodegradation reactions were performed in the air.

The photocatalytic reduction of CO_2 was carried out in a closed gas circulation system (quartz reactor) containing 100 ml of ultrapure water. First of all, the ZnO nanowire film was immersed into the water. Subsequently, high purity CO_2 gas was continuously bubbled into the above system for 20 min, in order to remove the air completely. With the irradiation of visible light, the amount of CO gas was measured by using a gas chromatograph (Agilent, GC-6820). During the test, the temperature of reactor was kept at 10 °C to increase the solubility of CO_2 by using a cooling water circulation.

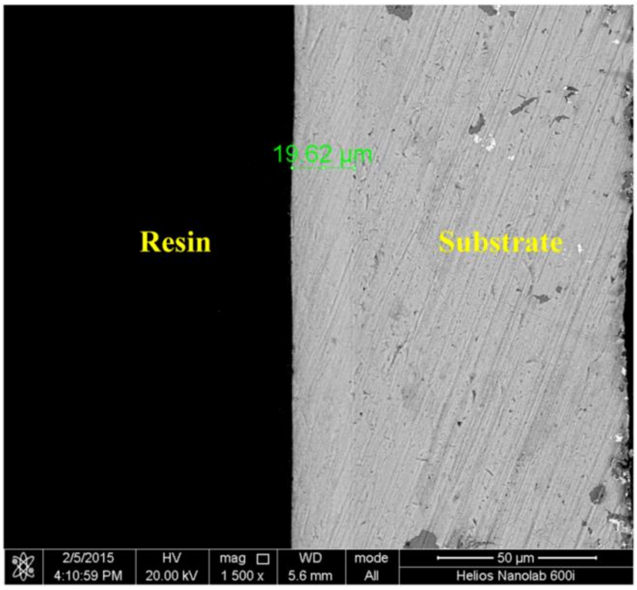

Figure S1. Cross-sectional morphology of nanocrystalline zinc coating.

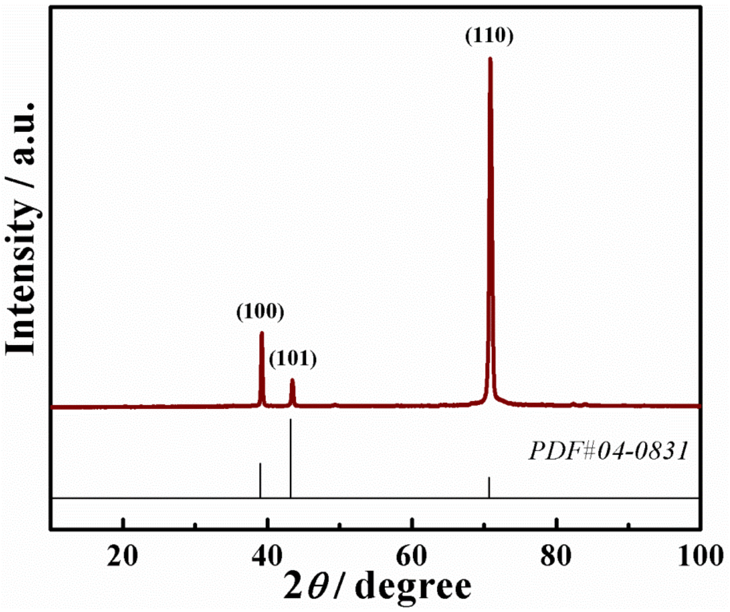

Figure S2. XRD spectrum of nanocrystalline zinc coating.

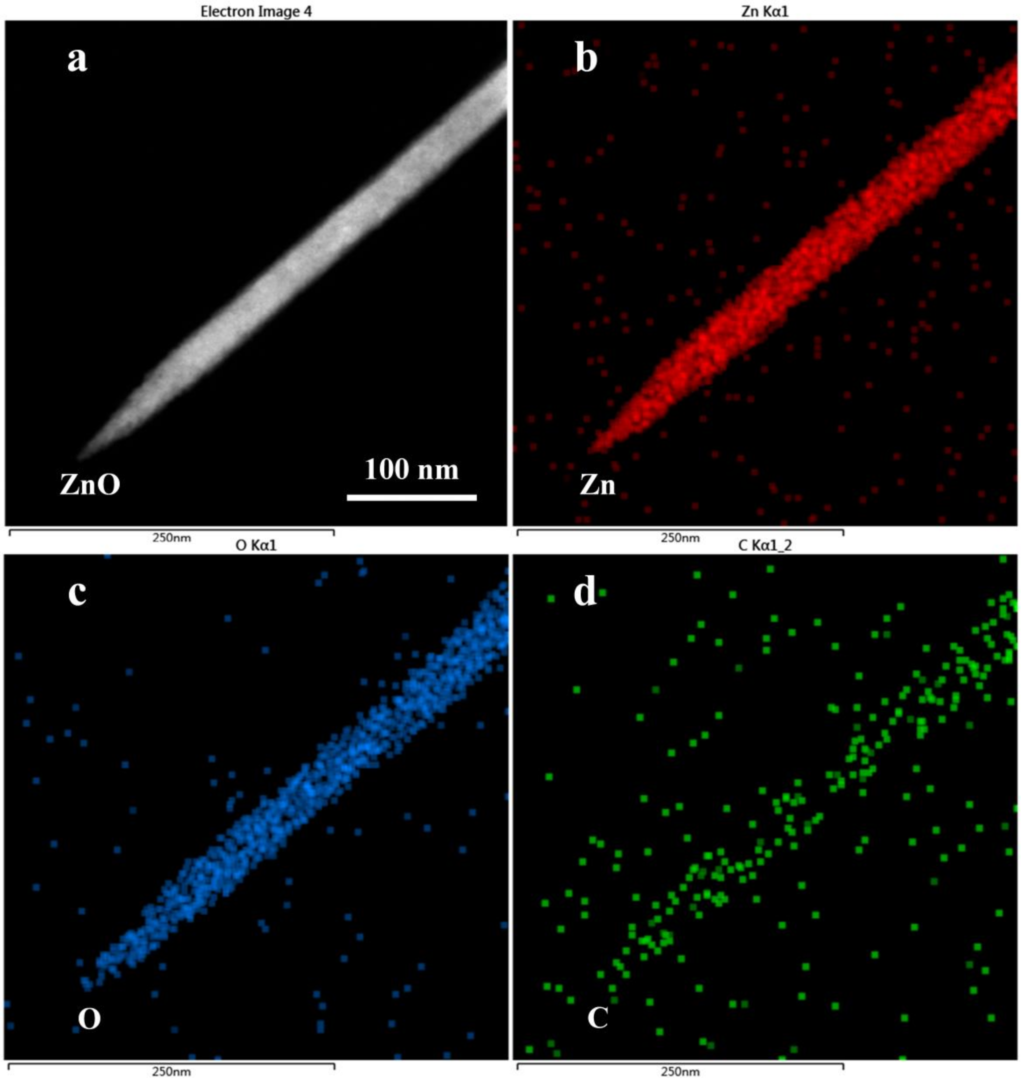

Figure S3. TEM image (**a**) and corresponding EDXS mappings (**b–d**) of single ZnO nanowire.

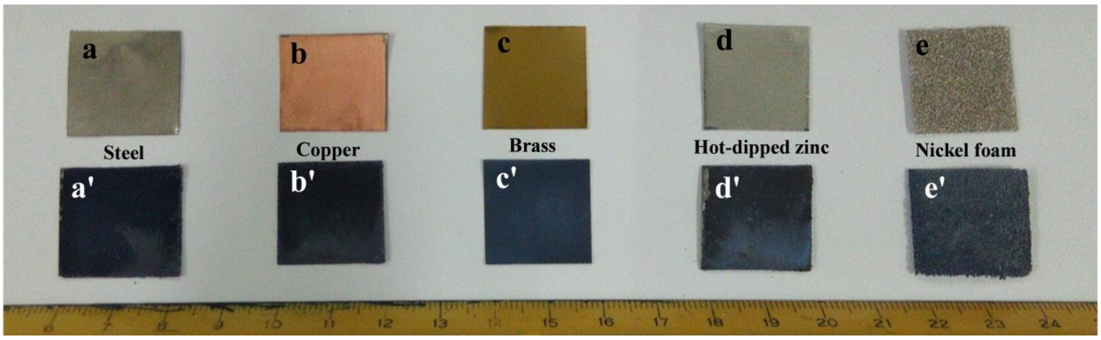

Figure S4. Digital photographs of steel, copper, brass, zinc as well as nickel substrates before (**a–e**) and after (**a'–e'**) coating of ZnO nanowire film.

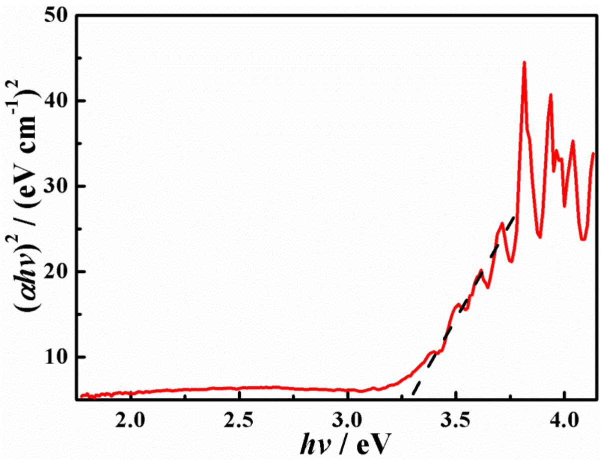

Figure S5. Variation of $(\alpha h\nu)^2$ versus the photon energy ($h\nu$) of ZnO nanowire film.

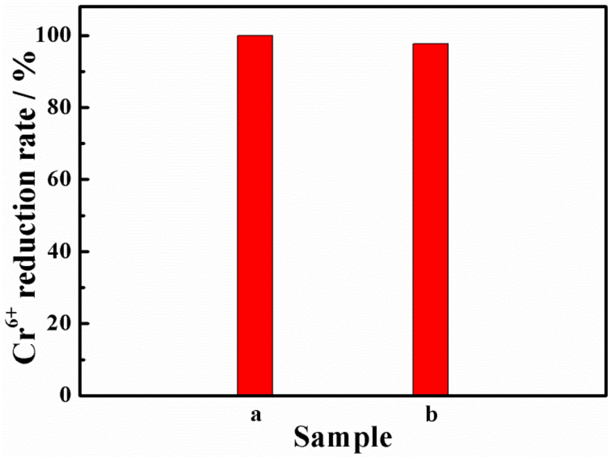

Figure S6. Comparison of the photocatalytic degradation rate between different Cr^{6+} solutions exposed to visible light for 140 min in the presence of ZnO nanowire film: (**a**) a 100 ml solution after seven continuous samplings (the sample interval is 5 ml every 20 min), and (**b**) a freshly prepared 100 ml solution.

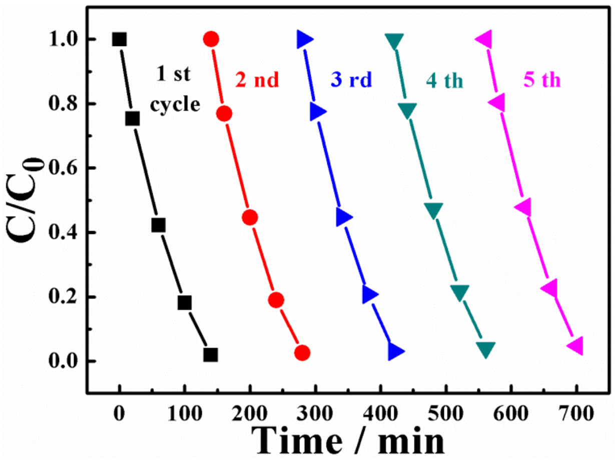

Figure S7. Photocatalytic activity of ZnO nanowire film for Cr^{6+} ions reduction with five separate cycles.

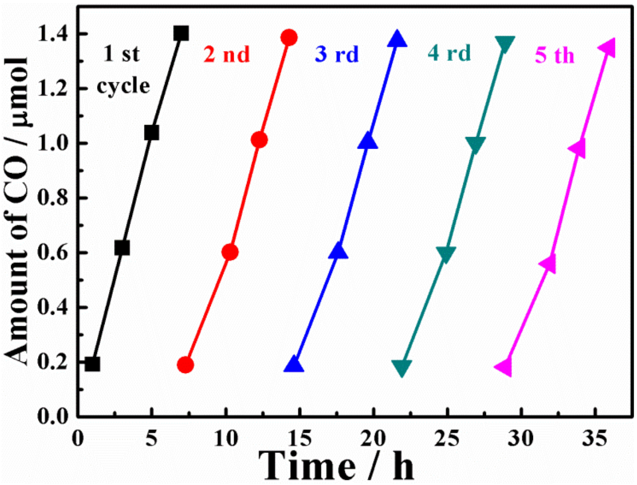

Figure S8. Cycling curves of photocatalytic CO_2 reduction for ZnO nanowire film.

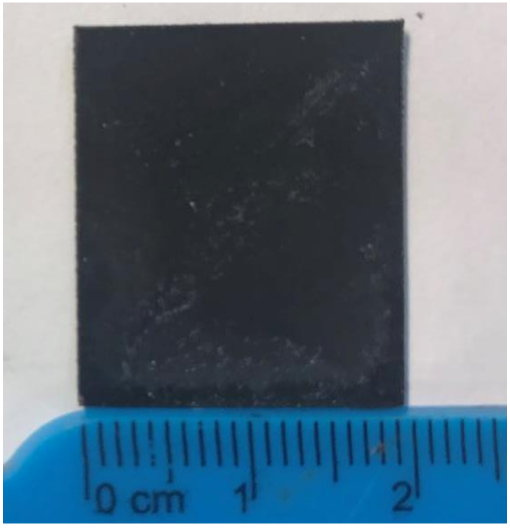

Figure S9. Digital photograph of ZnO nanowire film after the test of CO_2 photoreduction.

Table S1. A summary for the elemental content of different samples.

Samples	Atomic percentage		Weight percentage	
	Zn	O	Zn	O
Nanocrystalline zinc coating	100	–	100	–
ZnO nanowire film	44.18	55.82	76.38	23.62

Table S2. The grain size and texture coefficient calculated from XRD spectrum of nanocrystalline zinc coating.

	Peak/2θ			Average
	(100) 38.99°	(101) 43.22°	(110) 70.63°	
Grain size (nm)	39.8	35.2	26.2	33.7
Texture coefficient	0.12	0.04	2.85	

Table S3. Comparison of the Cr^{6+} ions reduction between ZnO nanowire film and other photocatalysts under visible light irradiation in literature.

Photocatalysts	Size	BET	m	$C_{Cr^{6+}}$	$V_{Cr^{6+}}$	Degradation rate	Ref.
	cm^2	m^2 g^{-1}	mg	mg L^{-1}	mL	mg min^{-1} cm^{-2} (or % min^{-1})	
ZnO nanowires film	5	-	-	10	100	1.429×10^{-3} (0.714)	This work
ZnO nanorods film	3.5	-	-	20	40	1.429×10^{-4} (0.063)	[1]
CuO microflower film	3.5	-	-	20	40	6.286×10^{-4} (0.275)	
ZnO nanoparticles powder	-	-	50	-	50	(0.142)	[2]
g-C_3N_4 microparticles powder	-	-	50	-	50	(0.075)	
SnS_2 nanoflakes powder	-	22.9	300	50	300	9.461×10^{-7} (0.433)	[3]
TiO_2 nanoparticles powder	-	134.9	300	50	300	4.015×10^{-8} (0.108)	
SnO_2 nanoparticles powder	-	145.5	300	50	300	0	[4]

References

[1] J. Yu, S. Zhuang, X. Xu, W. Zhu, B. Feng, J. Hu, Photogenerated electron reservoir in hetero-p–n CuO-ZnO nanocomposite device for visible-light-driven photocatalytic reduction of aqueous Cr (VI), *J. Mater. Chem. A* 3 (2015) 1199-1207.

[2] X. Yuan, C. Zhou, Q. Jing, Q. Tang, Y. Mu, A. K. Du, Facile synthesis of g-C3N4 nanosheets/ZnO nanocomposites with enhanced photocatalytic activity in reduction of aqueous chromium (VI) under visible light, *Nanomaterials* 6 (2016) 173.

[3] Y. C. Zhang, J. Li, H. Y. Xu, One-step in situ solvothermal synthesis of SnS2/TiO2 nanocomposites with high performance in visible light-driven photocatalytic reduction of aqueous Cr (VI), *Appl. Catal. B: Environ.*123 (2012) 18-26.

[4] Y. C. Zhang, L. Yao, G. Zhang, D. D. Dionysiou, J. Li, X. Du, One-step hydrothermal synthesis of high-performance visible-light-driven SnS2/SnO2 nanoheterojunction photocatalyst for the reduction of aqueous Cr (VI), *Appl. Catal. B: Environ.* 144 (2014) 730-738.
